# Polyphyllin VII induces apoptosis in HepG2 cells through ROS-mediated mitochondrial dysfunction and MAPK pathways

**DOI:** 10.1186/s12906-016-1036-x

**Published:** 2016-02-09

**Authors:** Chao Zhang, Xuejing Jia, Jiaolin Bao, Shenghui Chen, Kai Wang, Yulin Zhang, Peng Li, Jian-Bo Wan, Huanxing Su, Yitao Wang, Zhinan Mei, Chengwei He

**Affiliations:** 1 State Key Laboratory of Quality Research in Chinese Medicine, Institute of Chinese Medical Sciences, University of Macau, N22-7038, Avenida da Universidade, Taipa, Macao, 999078 China; 2 College of Pharmacy, South Central University for Nationalities, Wuhan, 430074 China

**Keywords:** Polyphyllin VII, Apoptosis, MAPK pathways, Anticancer, HepG2 cells

## Abstract

**Background:**

*Paris polyphylla* is an oriental folk medicine that has anticancer activities both in vivo and in vitro. Polyphyllin VII (PP7), a pennogenyl saponin from *P. polyphylla* has been found to exert strong anticancer activity. However, the underlying mechanisms are poorly understood. In the present study, the anticancer effect of polyphyllin VII against human liver cancer cells and the molecular mechanisms were investigated.

**Methods:**

Cellular viability was measured by MTT assay. Apoptosis, intracellular reactive oxygen species (ROS) and mitochondrial membrane potential levels were evaluated using the InCell 2000 confocal microscope. The expression levels of apoptotic-related proteins were evaluated by Western blotting.

**Results:**

PP7 strongly inhibited the cell growth and induced apoptosis and necrosis in hepatocellular carcinoma HepG2 cells. Meanwhile, PP7 up-regulated the levels of Bax/Bcl-2, cytochrome *c*, the cleaved forms of caspases-3, -8, -9, and poly (ADP-ribose) polymerase in a dose- and time-dependent manner, indicating that PP7 induced apoptosis in HepG2 cells through both intrinsic and extrinsic pathways. Moreover, PP7 provoked the production of intracellular ROS and the depolarization of mitochondrial membrane potential. Further analysis showed that PP7 significantly augmented the phosphorylation of JNK, ERK and p38, the major components of mitogen-activated protein kinase (MAPK) pathways, and the expressions of tumor suppressor proteins p53 and PTEN. In addition, PP7-induced apoptosis was remarkably attenuated by MAPK inhibitors and ROS inhibitor.

**Conclusions:**

These results demonstrated that PP7 induced apoptotic cell death in HepG2 cells through both intrinsic and extrinsic pathways by promoting the generation of mitochondrial-mediated ROS and activating MAPK and PTEN/p53 pathways.

**Electronic supplementary material:**

The online version of this article (doi:10.1186/s12906-016-1036-x) contains supplementary material, which is available to authorized users.

## Background

Cancer is one of the leading causes of death in the world and affects millions of patients worldwide despite significant advances have been made in the prevention, diagnosis, and treatment of cancers in recent years [1]. Hepatocellular carcinoma (HCC) is one of the most common cancers worldwide, ranging from 500,000 to 1 million new cases every year. Liver transplantation and surgical resection are the most efficient therapeutic strategies for early stage of HCC. However, current conventional therapies are still not effective to treat patients of advanced HCC [2]. Hence, it is imperative to develop novel therapeutic drugs with high efficiency and low toxicity for HCC. In recent years, traditional Chinese medicine (TCM) has been increasingly applied in HCC prevention and treatment due to the advantages of safety and lower drug resistance [3, 4]. Studies have shown that TCM possessed prophylactic and curative effects on human HCC with multiple actions and mechanisms [4, 5].

There are two major types of cell death: necrosis and apoptosis. Necrotic cell death is commonly a result of pathological process and often stimulates local or systemic inflammation. In contrast, apoptosis, a programmed cell death, is usually a physiological event that does not provoke inflammation [6]. Therefore, apoptosis was considered a potent therapeutic target for cancer treatment to reduce possible adverse side effects [7]. Numerous studies have demonstrated that apoptosis can be triggered by diverse pro-apoptosis stimuli converging on mitochondria, causing mitochondrial depolarization, cytochrome *c* release, caspase enzymes activation and eventually apoptotic cell death [8]. A number of signaling pathways including caspase cascade and mitogen-activated protein kinase (MAPK) pathways play a vital role in the process of apoptosis. Additionally, tumor suppressor proteins, such as phosphatase and tensin homolog (PTEN) and p53, are also important in promoting apoptosis. PTEN is one of the most frequently mutated, deleted, or silenced tumor suppressor genes in cancers. It regulates p53 transcriptional activity and protein levels [9]. Studies indicated that p53 induced apoptosis by either increasing transcriptional activity of pro-apoptotic genes or suppressing the activity of the anti-apoptotic genes [10]. Many findings have demonstrated that PTEN and p53 played a critical role in DNA damage response and regulated cell cycle and apoptosis [11, 12].

Recently, a number of natural steroidal saponins isolated from herbs display potential roles as apoptosis-promoting agents in a number of cancer cells [13–15], which have received increased attentions due to their unique properties. Pro-oxidant-induced apoptosis results in an increase in intracellular reactive oxygen species (ROS) formation, which was closely coupled with a number of downstream events in apoptosis. It is well known that relatively high level of ROS induces redox imbalance, causes cell apoptosis or necrosis during physiological and pathological progress of many diseases. Tumor cells with higher level of ROS tend to be killed more easily than normal cells with lower level of ROS. Therefore, we could explore new anticancer drugs with high potential in promoting ROS production. Some steroidal saponins have been reported to exert pro-oxidant actions [16, 17], which may be an important mechanism for their anticancer and apoptosis-inducing properties.

The rhizome of *Paris polyphylla* var. *yunnanensis*, or called *Rhizoma Paridis*, a long been used herb in TCM, possesses multiple pharmacological activities [18]. Notably, it has been used in combination with other herbs to treat cancer in clinical application of TCM [19]. Several saponins with significantly biological activities have been isolated and identified from *Rhizoma Paridis* and the steroidal saponins have been reported to have anticancer effects on many human cancer cell lines [20, 21].

Polyphyllin VII (PP7), one of the steroidal saponins from *Paris polyphylla*, has been found to show strong anticancer activity, as indicated by suppressing the growth of human cervical cancer HeLa cells [22] and colorectal cancer cells [23]. However, the underlying mechanisms of its anticancer activity are largely unknown. In this study, we aimed to investigate the anticancer effect of PP7 on human liver cancer HepG2 cells and the underlying molecular mechanisms. We found that PP7 induced apoptosis in HepG2 cells by inducing mitochondrial-mediated ROS generation and activating MAPK and PTEN/p53 pathways.

## Methods

### Reagents and antibodies

Polyphyllin II, VI and VII were kindly provided by Prof. Zhinan Mei (South-Central University for Nationalities). Dulbecco’s modified Eagle’s medium (DMEM) and Roswell Park Memorial Institute (RPMI) 1640 were obtained from Gibco (Carlsbad, CA, USA). Fetal calf serum (FCS) was obtained from Tianhang Biotech (Hangzhou, Zhejiang, China). Annexin V-fluorescein isothiocyanate (FITC) Apoptosis Detection Kit, mitochondrial membrane potential assay kit 5,5′,6,6′-tetra-chloro-1,1′,3,3′-tetra-ethyl-benzimidazol-carbocyanine iodide (JC-1), Hoechst 33342 staining kit, SP600125, SB203580, and PD98059 were purchased from Beyotime (Nanjing, Jiangsu, China). 3-(4,5-dimethylthiazol-2-yl)-2,5-diphenyltetrazolium bromide (MTT) was obtained from Molecular Probes (Eugene, OR, USA). 2′,7′-dichlorodihydrofluorescein diacetate (DCFH-DA) and N-acetyl-_L_-cysteine (NAC) were purchased from Sigma-Aldrich Co. LLC (Shanghai, China). Primary antibodies against Bcl-2, Bax, cytochrome *c*, p-BAD, BAD, p-stat3, stat3, p-PTEN, PTEN, extracellular signal-regulated kinase (ERK), p-ERK, p38 MAPK (p38), p-p38, c-Jun N-terminal kinase (JNK), p-JNK, p53, cleaved caspase-3, -8, -9 and poly (ADP-ribose) polymerase (PARP) and glyceraldehyde-3-phosphate dehydrogenase (GAPDH), and secondly antibodies were purchased from Cell Signaling Technology (Danvers, MA, USA) or Proteintech Group Inc (Wuhan, Hubei, China). The enhanced chemiluminescence (ECL) detection kit was obtained from Amersham Pharmacia Biotech (Buckinghamshire, UK). All other chemicals of analytical grade were purchased from local sources.

### Cell culture and treatments

Human hepatocellular carcinoma cell lines HepG2, Hep3B and Bel7402, human lung carcinoma cell line A549, human breast adenocarcinoma cell line MCF-7, human colorectal adenocarcinoma cell line Caco-2, and human ovarian carcinoma cell line SKOV-3 were obtained from American Type Culture Collection (Manassas, VA, USA). Human hepatocellular carcinoma cell line SMMC-7721 was obtained from the Cell Bank of Shanghai Institute of Biochemistry and Cell Biology, Chinese Academy of Sciences (Shanghai, China). Cells were grown as monolayers in RPMI 1640 or DMEM. Cells were supplemented with 10 % heat-inactivated FCS, and 100 units/mL penicillin-streptomycin (Caco-2 cells plus 4 mM glutamine and 1 % nonessential amino acids), and incubated at 37 °C in a humidified atmosphere containing 5 % CO_2_. For all in vitro assays, PP7 was dissolved in dimethyl sulfoxide (DMSO) to make a stock solution, sterilized using a sterile 0.22 μm membrane filter and stored at -80 °C, at a final DMSO concentration of less than 0.1 %. The working solutions were freshly diluted in the basal medium.

### Cell viability assay

The cytotoxicity of PP7 was measured using MTT colorimetric assay. Briefly, cells (1 × 10^4^ cells/well) were seeded in 96-well plates. After complete adhesion, different dilutions of PP7 were added and incubated further for 24, 48 and 72 h. The treated cells were then incubated in freshly DMEM medium containing MTT (0.2 mg/mL) at 37 °C. After 4 h, the supernatants were discarded carefully and added DMSO to dissolve the formazan crystals. The absorbance at 570 nm was measured with a microplate reader (BioTek, Winooski, VT, USA). The relative viability of treated cells was expressed as percentage of control untreated cells.

### Determination of apoptosis and necrosis

Apoptosis was determined by visualizing surface exposure of phosphatidylserine using annexin V-FITC [24]. HepG2 cells were either treated with different concentrations of PP7 or left untreated as control for 24 h. Cells were then resuspended in 500 μL binding buffer and incubated with FITC-conjugated annexin V (0.25 μg/mL), Hoechst 33342 (10 μg/mL) and propidium iodide (3 μg/mL)/RNase (30 μg/mL) for 15 min at 37 °C in the dark. After supravital staining, the cells were immediately visualized using the InCell 2000 confocal microscope (GE Biosciences, Piscataway, NJ, USA). Each well was taken at least six images (original magnification × 20). Images were captured by visualizing blue fluorescence (Hoechst 33342), green fluorescence (Annexin-V) and red fluorescence (propidium iodide). The number of positive cells per well was counted using the software modules supplied with the InCell 2000 and was expressed relative to the total number of nuclei present.

### Measurement of intracellular reactive oxygen species (ROS)

The intracellular ROS generation was detected using an oxidant sensitive fluorescent probe DCFH-DA. To test the effect of time-dependent ROS generation, HepG2 cells seeded in 96-well plates (1 × 10^4^/well) were treated with PP7 at the concentration of 1.32 μM for 3, 6, 12 and 24 h, respectively. To test the effect of concentration-dependent ROS generation, cells were treated with PP7 of 0.59, 0.88, 1.32 and 1.98 μM for 24 h. After treatment with DCFH-DA for 30 min at 37 °C in the dark, the cells were washed three times with phosphate buffered saline and fluorescence images of the cells were acquired using the InCell 2000 confocal microscope. For quantitative evaluation of intracellular ROS production efficacy, the fluorescence was measured by the software modules supplied with the InCell 2000.

### Measurement of mitochondrial membrane potential (ΔΨm)

The mitochondrial membrane potential was measured using JC-1, which is a mitochondria-specific lipophilic cationic fluorescence dye and is capable of selectively entering the mitochondria. HepG2 cells were plated in a 96 well plate at a density of 1 × 10^4^ cells/well and grown for 24 h. Different concentrations of PP7 were added and incubated further for 24 h. HepG2 cells were then stained with JC-1 working solution for 20 min at 37 °C in the dark, washed twice with 100 μL JC-1 binding buffer, and placed in growth media. Plates were then imaged live with the InCell 2000 confocal microscope. Quantitative image analysis was carried out using the software modules supplied with the InCell 2000.

### Determination of the expression levels of apoptotic- and MAPK-Related Proteins

For Western blotting analysis, HepG2 cells of different treatment groups were collected. Proteins were extracted with radio immunoprecipitation assay lysis buffer and separated using 8-12 % sodium dodecylsulfate polyacrylamide gel electrophoresis, and electrotransferred to polyvinylidene fluoride membranes. The membranes were blocked with 5 % non-fat milk and incubated overnight at 4 °C with primary antibodies followed by incubation with the corresponding secondary antibodies. Signals were developed using an ECL detection kit according to the manufacturer’s instructions. Densitometric measurement of band intensity was performed with Image Lab Software (Bio-Rad, Hercules, CA, USA). The expression of GAPDH was used as an internal standard.

### Inhibitor treatment

To clarify the roles of signaling pathways in PP7-induced apoptosis in HepG2 cells, the cells were pretreated with the following inhibitors individually before the treatment of PP7 (1.32 μM): 20 μM SP600125 (JNK specific inhibitor), 5 μM PD98059 (ERK specific inhibitor), 20 μM SB203580 (p38 specific inhibitor), or 10 μM NAC (a free radical scavenger). The cells were then subjected to the determination of apoptosis by annexin V-FITC staining and measurement of apoptosis-related protein expression by Western blotting as described above.

### Statistical analysis

All data were given as the means ± standard deviation (SD) for at least three independent experiments, and analyzed for statistical significance using one-way analysis of variance, followed by Tukey post hoc analysis using GraphPad Prism 5 software package (GraphPad Software, San Diego, CA, USA). A *p*-value < 0.05 was considered to be statistically significant.

## Results

### Cytotoxic effect of PP7 on human cancer cells

To examine the cytotoxicity of PP7, five human cancer cell lines (MCF7, Caco2, SKOV3, A549 and HepG2) were exposed to PP7 at different concentrations for 24 h before MTT assay. The results showed that PP7 significantly inhibited the growth of all cell lines, with 50 % inhibitory concentration (IC_50_) values in the range from 1.32 to 2.70 μM. HepG2 showed the highest sensitivity to PP7 among these five cancer cell lines, with an IC_50_ value of 1.32 ± 0.04 μM (Additional file 1). We also tested the cytotoxic effects of other two steroidal saponins from *Paris polyphylla*, Polyphyllin II (PP2) and Polyphyllin VI (PP6). Both of them exhibited strong cytotoxic activity against a panel of human cancer cells (Additional files 2 and 3). Comparing to PP2 and PP6, PP7 demonstrated the highest cytotoxicity on the tested cell lines. Therefore, we further investigated the anticancer effects of PP7 on liver cancer cells and the underlying mechanisms.

Liver cancer cell lines HepG2, Hep3B, Bel7402 and 7721 cells were treated with PP7 at concentrations from 0.17 μM to 10 μM for 24, 48, and 72 h. As indicated in Table 1, PP7 showed the highest cytotoxicity against HepG2 cells than the other liver cancer cells, suggesting its potential selective cytotoxicity to cancer cell lines. In addition, prolonged exposure of these cells to PP7 resulted in an increased growth inhibitory effect, indicating that the *in vitro *anticancer activity of PP7 was in a time-dependent manner (Table 1). Since HepG2 cell line was the most sensitive to the treatment of PP7 among the tested liver cancer cell lines, we chose HepG2 cell line for the subsequent study.

**Table 1:** Cytotoxicity of Polyphyllin VII (PP7) against human liver cancer cells (HepG2, Hep3B, Bel7402 and 7721). Cells were treated with increasing concentrations of PP7 for 24, 48 and 72 h and cell viability was determined by MTT assay as described in Methods section. Each IC_50_ value represents means ± SD of 3 to 5 independent experiments

Cell line		IC_50_ (μM)	
	24 h	48 h	72 h
HepG2	1.32 ± 0.04	0.85 ± 0.03	0.78 ± 0.04
Hep3B	2.61 ± 0.08	1.84 ± 0.06	1.23 ± 0.05
Bel7402	2.86 ± 0.15	1.74 ± 0.06	1.12 ± 0.04
7721	2.30 ± 0.09	1.73 ± 0.05	1.28 ± 0.05

### PP7 induced apoptosis and necrosis in HepG2 cells

**Fig. 1:**
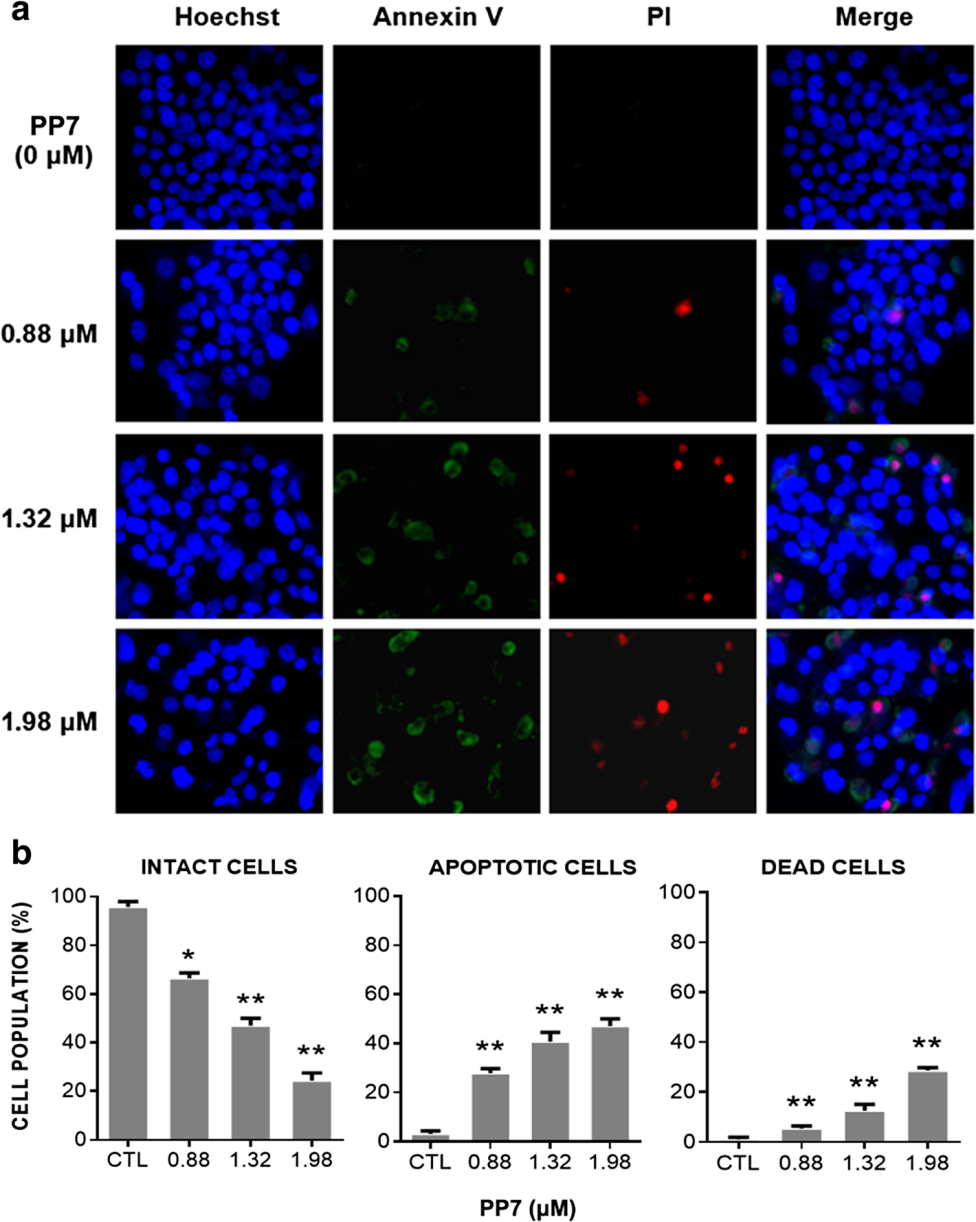
Polyphyllin VII (PP7) induced apoptosis and necrosis in HepG2 cells. **a** HepG2 cells were treated with indicated concentrations of PP7 for 24 h and photographed using the InCell 2000 confocal microscope after Hoechst 33342 (blue), Annexin V (green) and PI (red) staining. **b** The number of cells displaying intact cells, apoptotic cells or dead cells was counted, * *p* < 0.05, ** *p* < 0.01, versus control (CTL). Data are represented as means ± SD from 3 independent experiments

We next investigate whether the cytotoxicity of PP7 on HepG2 cells is related to the induction of apoptosis and necrosis. Apoptosis and necrosis were determined by Hoechst/Annexin-V/PI triple staining and InCell 2000 confocal microscope after treatment of HepG2 cells with PP7 for 24 h. Characteristic examples of our observations and quantitative image analysis are shown in Fig. 1a and b. As for the apoptotic cells, they can be stained with Annexin-V-FITC and were characterized by a green fluorescence in PP7-treated cells in a dose-dependent manner (Fig. 1a). The percentage of apoptosis in HepG2 cells treated with 0.88, 1.32 and 1.98 μM of PP7 was 13.3, 19.2 and 40.0 %, respectively (Fig. 1b). Necrotic cells can be stained by PI with a red fluorescence were observed in Fig. 1a. The percentage of necrosis in HepG2 cells was 5.6, 12.5 and 28.7 %, individually corresponding to 0.88, 1.32 and 1.98 μM of PP7 treatment (Fig. 1b). Meanwhile, HepG2 cells treated with various concentrations of PP7 presented different degrees of cell growth inhibition detected by a blue fluorescence staining of nuclei with Hoechst 33342, which was consistent with the result shown in Table 1.

### Effects of PP7 on mitochondrial membrane potential (ΔΨm) and intracellular ROS in HepG2 cells

**Fig. 2:**
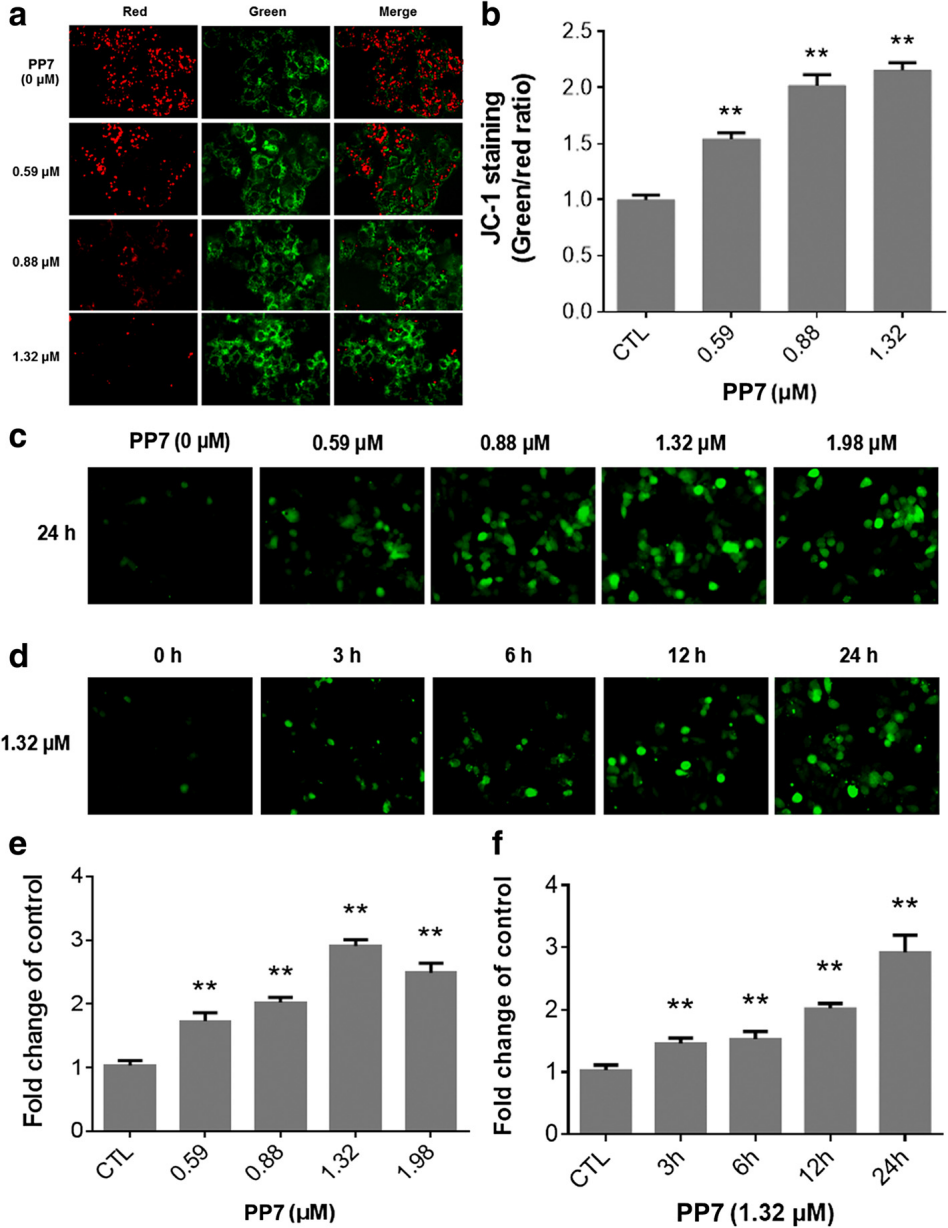
Effects of Polyphyllin VII (PP7) on mitochondrial membrane potential and intracellular reactive oxygen species (ROS) in HepG2 Cells. **a** HepG2 cells were treated with indicated concentrations of PP7 for 24 h and photographed using the InCell 2000 confocal microscope after JC-1 staining. **b** Quantitative analysis of green (FITC)/red (CY3) fluorescence in HepG2 cells, ** *p* < 0.01, compared to control (CTL). Observation of intracellular ROS generation by DCFH-DA staining in HepG2 cells after treated with indicated doses of PP7 for 24 h (**c**, **e**) or treated with 1.32 μM PP7 for different times (**d**, **f**). ** *p* < 0.01, compared to CTL. Data are both represented as means ± SD from 3 independent experiments

JC-1 staining was applied to detect changes in the mitochondrial membrane potential of HepG2 cells. As shown in Fig. 2a, a red fluorescence represents normal mitochondria in vehicle-treated cells, whereas a green fluorescence represents depolarized mitochondria in PP7-treated cells. The ratios of JC-1 green-to-red fluorescence intensities were increased by 54.2, 101.6 and 115.3 % when HepG2 cells were treated with 0.59, 0.88 and 1.32 μM of PP7 for 24 h (Fig. 2b). These results revealed that PP7 could increase mitochondrial membrane permeability and cause mitochondrial dysfunction of HepG2 cells, indicating that the induction of apoptosis in HepG2 cells by PP7 was through the mitochondrial pathway.

Several studies have shown that the apoptosis induced by saponins is associated with ROS generation in cells [16, 17]. Therefore, we next determine whether PP7 could promote ROS generation in HepG2 cells. DCFH-DA would be hydrolyzed to dichlorofluorescein (DCF) in the cell and yield a highly green fluorescent signal in the presence of intracellular ROS. Quantitative analysis found that PP7 could rapidly increase the fluorescence intensity, indicating an increase in ROS level in HepG2 cells compared with the control group (Fig. 2e and f). The mean intracellular DCF fluorescence intensity was increased by 190.5 % in cells treated with 1.32 μM of PP7 for 24 h (Fig. 2c and e). A significant increase in ROS was observed at 3 h, while a peak value appeared at 24 h after PP7 treatment (Fig. 2d and f). To further determine the role of ROS production in the process of apoptosis in HepG2 cells induced by PP7, the cells were pretreated with 10 μM NAC (a free radical scavenger) for 2 h and subsequently treated with 1.32 μM PP7 for 24 h. The induction of apoptosis and necrosis was assessed by Hoechst/Annexin-V/PI triple staining and InCell 2000 confocal microscope. Figure 5b and c showed that pretreatment with NAC significantly decrease the apoptosis and necrosis in PP7-treated HepG2 cells. These results indicated that PP7 caused oxidative stress in HepG2 cells and ROS production plays an important role in PP7-induced apoptosis.

### Effects of PP7 on the expression of apoptosis-related proteins in HepG2 cells

**Fig. 3:**
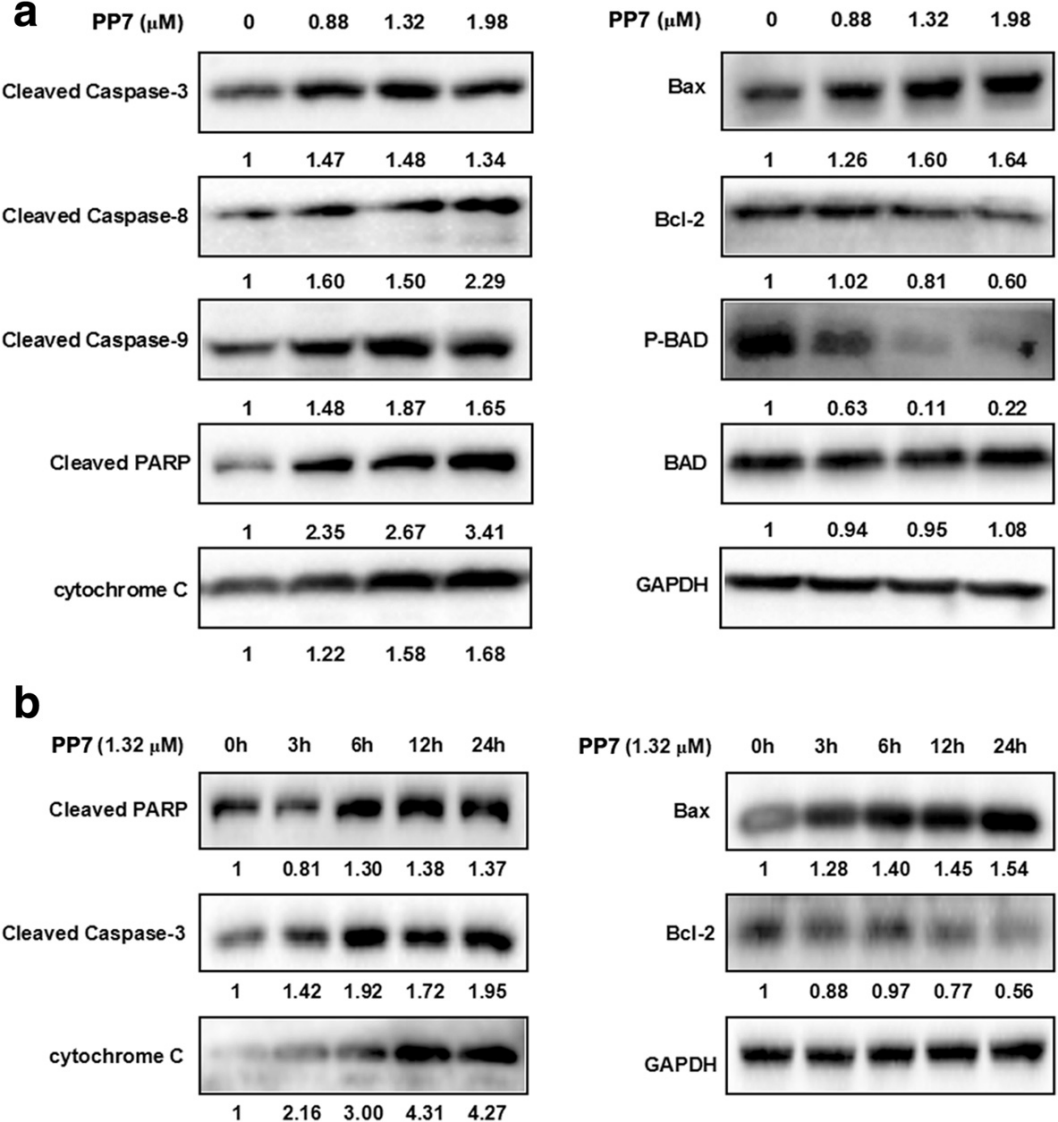
Effects of Polyphyllin VII (PP7) on the expression of apoptosis-related proteins in HepG2 cells. HepG2 cells were treated with PP7 in the indicated concentrations for 24 h (**a**) or treated with 1.32 μM PP7 for the indicated times (**b**). The protein levels of apoptosis-related proteins were determined by Western blotting as described in Methods sections. The indicated number under each band was the fold changes of expression level compared to that in the control group. GAPDH was used as the internal standard for protein loading

In order to explore the mechanisms underlying PP7 induced apoptosis, the expression of apoptosis-related proteins in HepG2 cells was investigated by Western blotting. Compared to the vehicle-treated control group, the expression level of cleaved caspases-3, -8, -9 and PARP increased by 34 %, 129 %, 65 % and 241 %, respectively, in HepG2 cells treated with 1.98 μM of PP7 for 24 h. Meanwhile, the expression of pro-apoptotic protein Bax and cytochrome *c* were increased while the anti-apoptotic protein Bcl-2 and phosphorylated Bcl2-associated agonist of cell death (BAD), an anti-apoptotic form of BAD protein, were suppressed by PP7. The effects of PP7 on the expression of apoptosis-related proteins in HepG2 cells were in an obvious time- and dose-dependent manner (Fig. 3). These data suggested that PP7 induced apoptosis in HepG2 cells through intrinsic and extrinsic apoptotic pathways depending on caspase activation.

### Effects of PP7 on the expression of PTEN, p53 and STAT3 in HepG2 cells

**Fig. 4:**
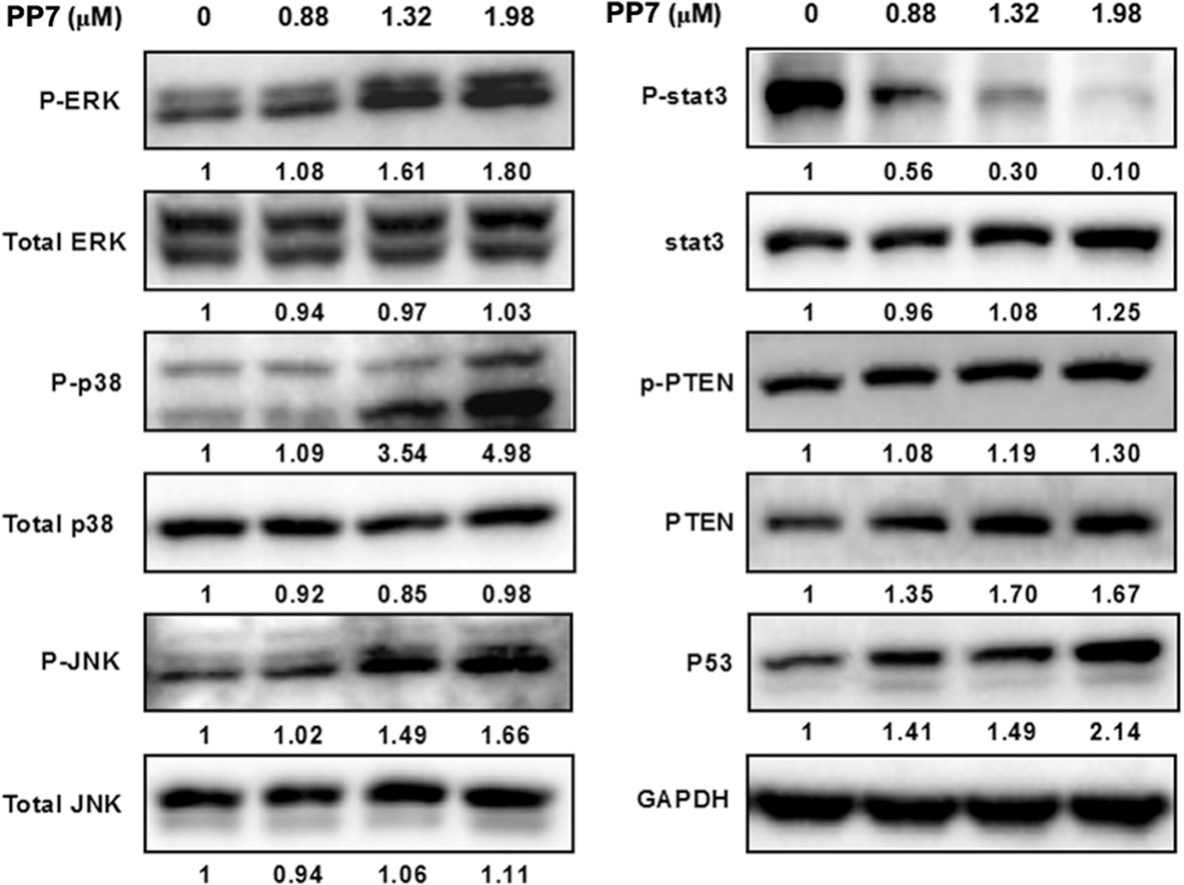
Effects of Polyphyllin VII (PP7) on the MAPK and PTEN/p53 pathways in HepG2 cells. Key proteins in MAPK pathways, PTEN and p53 were determined by Western blotting analysis after treating the cells with PP7 at indicated concentrations for 24 h. The indicated number under each band was the fold changes of expression level compared to that in the control group. GAPDH was used as the internal standard for protein loading

We next tested whether the tumor suppressors PTEN and p53 and their potential downstream target STAT3 [11, 12, 25] were involved in PP7-induced apoptosis in HepG2 cells. As shown in Fig. 4, total protein levels of PTEN and p53 were increased by 67 % and 114 %, while the phosphorylation of STAT3 decreased by 90 % in HepG2 cells exposed to 1.98 μM of PP7 for 24 h, suggesting that PP7-induced apoptosis in HepG2 cells might be mediated through upregulation of PTEN and p53 expression, and downregulation of STAT3 activation.

### PP7 induces apoptosis of HepG2 cells via MAPK pathways

**Fig. 5:**
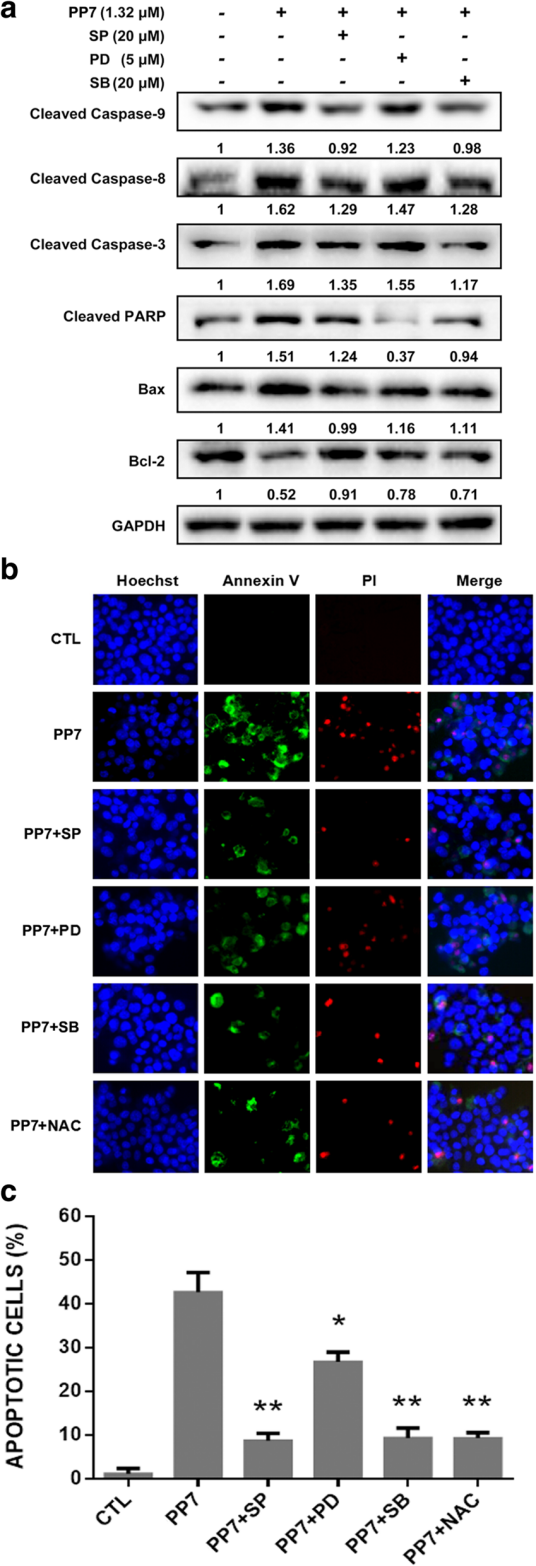
Inhibition of MAPK pathways and ROS production attenuated PP7-induced apoptosis and necrosis in HepG2 cells. **a** Effects of JNK inhibitor SP600125, ERK inhibitor PD98059 and p38 inhibitor SB203580 on the expression of apoptosis-related proteins in PP7-treated cells by Western blotting analysis. The indicated number under each band was the fold changes of expression level compared to that in the control group. GAPDH was used as the internal standard for protein loading. **b** Effects of SP600125 (SP) , PD98059 (PD), SB203580 (SB) and NAC on PP7-induced apoptosis and necrosis tested by Hoechst 33342, Annexin V and PI staining. **c** Quantification of apoptotic cells in (**b**). * *p* < 0.05, ** *p* < 0.01, versus control (CTL). Data are represented as means ± SD from 3 independent experiments

As shown in Fig. 4, the phosphorylation of JNK, ERK and p38, the major components of MAPK signaling pathways, was significantly increased in HepG2 cells treated with PP7 in a dose-dependent manner. To further exam the role of MAPKs in PP7-induced apoptosis in HepG2 cells, JNK inhibitor SP600125 (20 μM), ERK inhibitor PD98059 (5 μM) and p38 inhibitor SB203580 (20 μM) were used to pretreat HepG2 cells before the treatment of PP7. As shown in Fig. 5a, MAPK inhibitors could significantly reduce the expression of apoptosis-related proteins in HepG2 cells and decrease the apoptosis and necrosis of HepG2 cells treated with PP7 (Fig. 5b and c). Together, these results indicated that MAPK signaling pathways might mediate PP7-induced apoptosis in HepG2 cells.

## Discussion

The steroidal saponins isolated from herbs possess structural diversity and significant anticancer activities [13–15]. In recent years, studies demonstrated that the main anticancer components of *Rhizoma Paridis* are steroidal saponins [20, 21]. Polyphyllin VII (PP7), a steroidal saponin purified from *P. polyphylla* has been identified as a potential anticancer agent in many pharmacological studies [22, 23]. However, the cellular and molecular mechanisms for the anticancer activity of PP7 are poorly understood. In the present study, we found that PP7 could significantly inhibit the proliferation of a panel of human cancer cell lines in a dose-dependent manner (Additional file 1). Among the tested cancer cell lines, HepG2 was the most sensitive to PP7 treatment (Table 1), indicating that PP7 exhibited relatively selective anticancer activity. Considerable studies have shown that natural compounds isolated from TCM were effective in inhibiting cancer cell proliferation of HCC [4]. Oridonin, from *Isodon rubescens*, inhibited the proliferation of HepG2 cells with an IC_50_ of 41.77 μM for 24 h [26]. The IC_50_ value of oroxylin A, from *Scutellaria baicalensis*, was 23.94 μM against HepG2 cells after 24 h treatment [27]. The IC_50_ of arsenic trioxide after exposure HepG2 cells for 72 h, was approximately 2.25 μM [28]. In the present study, PP7 significantly inhibited the growth of HepG2 cells, with an IC_50_ value as low as 1.32 μM for 24 h treatment, indicating that PP7 exhibits strong cytotoxicity against HepG2 cells. Induction of apoptosis is regarded as a novel therapeutic strategy for cancers [6]. Many anticancer agents have been reported to induce cell death of tumor cells by triggering apoptosis [29]. In our results, PP7 strongly induced apoptotic cell death in HepG2 cells in a dose-dependent manner as evidenced by Annexin V/Hoechst 33342 staining (Fig. 1), suggesting that the anticancer effect of PP7 on HepG2 cells was mediated through the induction of apoptosis.

Various chemotherapeutic agents exert their anticancer effects through inducing the generation of ROS, and the intrinsic apoptotic pathway is especially susceptible to ROS. In the present study, the production of intracellular ROS increased remarkably in HepG2 cells treated with PP7 (Fig. 2c and d), while inhibition of ROS production by the antioxidant NAC significantly decreased the apoptosis (Fig. 5b), suggesting that PP7-induced apoptosis in HepG2 cells was closely associated with the production of ROS, which may act as upstream signaling molecules to initiate mitochondria-mediated cell apoptosis. ROS is involved in the opening of the mitochondrial permeability transition pore, depolarization of the mitochondrial membrane, and then the release of mitochondrial pro-apoptotic factors in the process of mitochondria-mediated apoptosis [30]. In our results, PP7 increased the ratio of JC-1 green-to-red fluorescence intensity (Fig. 2a), indicating a decreased ΔΨm and depolarization of the mitochondrial membrane, further confirmed that PP7 induced an ROS-involved mitochondrial pathway-mediated apoptosis in HepG2 cells. Similar results were reported that berberine, an isoquinoline alkaloid isolated from TCM, exhibited cytotoxicity on L929 murine fibroblast cells was related to accumulation of intracellular ROS, reduction of mitochondrial membrane potential, and cell apoptosis [31].

Apoptosis is executed through mitochondrial-mediated intrinsic and cell death receptor-mediated extrinsic pathways, both of which are converged to the cascade activation of caspase proteases [32]. In the intrinsic pathway, cytochrome *c* releases from damaged mitochondria and causes apoptosome-dependent activation of caspase 9. This leads to the activation of the executioner caspases 3 [33], and eventually inactivation of PARP [30]. In the extrinsic pathway, activated initiator caspase-8 by death-inducing signaling complex also cleaves and activates caspase-3 and results in apoptotic cell death [34]. Our results demonstrated that PP7 increased the levels of cleaved caspases-3, -8, -9 and PARP in a dose- and time-dependent manner (Fig. 3), indicating that both intrinsic and extrinsic pathways were involved in the process of PP7-induced apoptosis in HepG2 cells. Our findings are consistent with the previous findings that TCM induced cancer cell apoptosis through intrinsic and extrinsic pathways [35, 36].

Increased Bax/Bcl-2 ratio leads to ΔΨ*m* collapse, cytochrome *c* release, caspase-3 activation, and eventually apoptosis [37]. In addition, pro-apoptotic proteins BAD and Bax are closely associated with the control of mitochondrial membrane permeability and release of cytochrome *c* [38]. Our results showed that PP7 increased the ratio of Bax/Bcl-2, decreased the level of p-BAD (an anti-apoptotic form), and caused the release of cytochrome *c* from mitochondria in a dose- and time-dependent manner (Fig. 3), further confirmed that mitochondrial pathway was involved in PP7-induced apoptosis in HepG2 cells.

Numerous reports have demonstrated that expression of apoptosis-related proteins is critically regulated by tumor suppressor proteins PTEN and p53 in response to DNA damage [11, 12]. Oxidative stress promotes PTEN nuclear accumulation, which leads to p53 stabilization and p53-mediated apoptosis [39]. Furthermore, ROS could cause DNA damage and then directly activate p53 and initiate apoptosis [40]. In the current study, PP7 significantly increased the expression levels of PTEN and p53 in HepG2 cells (Fig. 4), suggesting their potential roles in PP7-induced apoptosis in HepG2 cells. The upregulation of PTEN and p53 expression could be promoted by increased intracellular ROS production in HepG2 cells by PP7, which is in line with previous reports [41–43].

MAPKs are also the major oxidative stress-sensitive signal transducing pathways [44] and serve as upstream signals for the initiation of apoptosis. JNK and p38 MAPK were activated in response to ROS generation and mitochondrial dysfunction, which are frequently associated with the induction of apoptosis [45]. Li et al. [46] reported that the total flavonoids from *Arachniodes exilis* induced apoptosis in HepG2 cells through ROS-mediated MAPK activation and mitochondrial dysfunction. A novel oleanolic acid derivative induces human hepatoma cell apoptosis via an ROS/MAPK-dependent mitochondrial pathway [47]. Our results showed that PP7 significantly increased the levels of phosphorylated JNK, ERK and p38 in a dose-dependent manner without affecting the expression of total proteins (Fig. 4), indicating that these MAPK pathways were activated in the process of PP7-induced apoptosis in HepG2 cells. Moreover, MAPK inhibitors decreased the apoptosis of HepG2 cells (Fig. 5) induced by PP7. These results demonstrated that activation of ERK, JNK, and p38 pathways were involved in PP7-induced apoptosis in HepG2 cells.

The transcription factor STAT3 promotes tumorigenesis mainly through preventing apoptosis by up-regulating the expression of apoptosis inhibitors in several primary human cancers [48], and blockage of constitutive STAT3 signaling results in the growth inhibition and apoptosis in cancer cells [49]. Studies reported that the constitutive activation of STAT3 was often negatively regulated by p53, PTEN, and MAPK pathways [25, 50]. Our data showed that PP7 significantly decreased the phosphorylated STAT3 level in HepG2 cells (Fig. 4). We supposed that STAT3 might be inhibited by the upregulated p53, PTEN, and MAPKs, in which the details need to be further elucidated.

## Conclusion

**Fig. 6:**
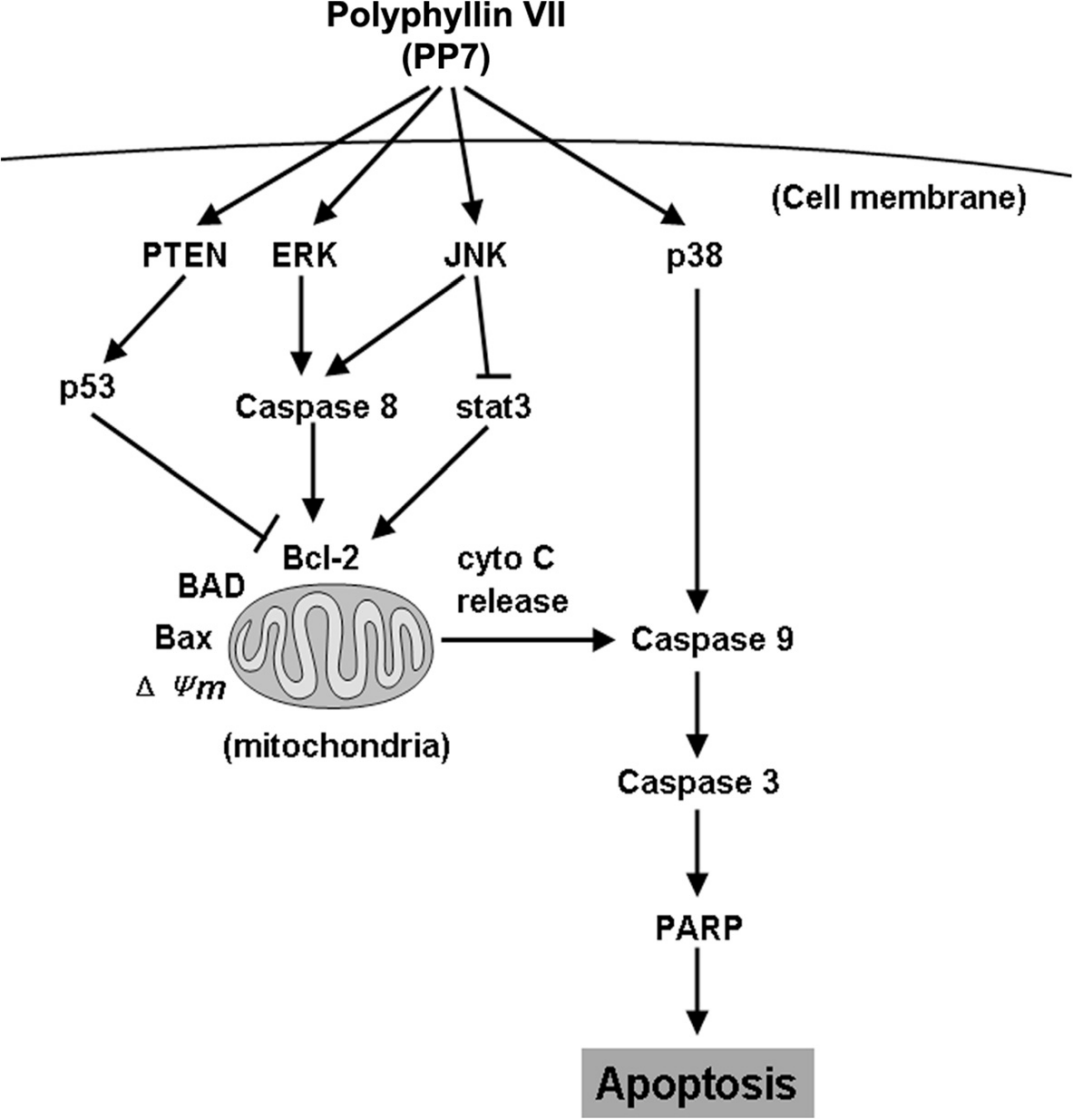
Schematic diagram showing the proposed apoptotic signaling pathways triggered by PP7 in HepG2 cells. PP7 treatment leads to the activation of MAPK (JNK, ERK, and p38) and PTEN/p53 signaling pathways, followed by upregulation of pro-apoptotic proteins and downregulation of anti-apoptotic protein, and eventually causes apoptosis in HepG2 cells

In summary, we demonstrated that PP7 induced apoptotic cell death in HepG2 cells through both intrinsic and extrinsic pathways via triggering mitochondrial-mediated ROS generation and activating MAPK and PTEN/p53 pathways (Fig. 6). This study provides an insight into the molecular mechanisms of PP7-induced apoptosis in liver cancer cells and presents that PP7 may be a novel promising agent for liver cancer treatment.

## Additional files

Additional file 1: **Polyphyllin VII (PP7) inhibited the proliferation of human cancer cells. Cells were treated with increasing concentrations of PP7 for 24 h and cell viability was determined by MTT assay as described in Methods sections.** Each IC_50_ value represents means ± SD of 3 to 5 independent experiments. (TIF 382 kb)

Additional file 2: **Polyphyllin II (PP2) inhibited the proliferation of human cancer cells (HepG2, A549, MCF7, SKOV3, Hep3B, Bel7402 and 7721). Cells were treated with increasing concentrations of PP2 for 24 h and cell viability was determined by MTT assay as described in Methods sections .** Each IC_50_ value represents means ± SD of 3 to 5 independent experiments. (TIF 593 kb)

Additional file 3: **Polyphyllin VI (PP6) inhibited the proliferation of human cancer cells (HepG2, A549, MCF7, SKOV3, Hep3B, Bel7402 and 7721).** Cells were treated with increasing concentrations of PP6 for 24 h and cell viability was determined by MTT assay as described in Methods sections. Each IC_50_ value represents means ± SD of 3 to 5 independent experiments. (TIF 594 kb)

## Abbreviations

HCC: hepatocellular carcinoma
MAPK: mitogen-activated protein kinase
PTEN: phosphatase and tensin homolog
ROS: reactive oxygen species
TCM: traditional Chinese medicine
PP7: polyphyllin VII
DMEM: Dulbecco’s modified Eagle’s medium
RPMI: Roswell Park Memorial Institute
FCS: fetal calf serum
FITC: fluorescein isothiocyanate
JC-1: 5,5′,6,6′-tetra-chloro-1,1′,3,3′-tetra-ethyl-benzimidazol-carbocyanine iodide
DCFH-DA: 2′,7′-dichlorodihydrofluorescein diacetate
NAC: N-acetyl-_L_-cysteine
ECL: enhanced chemiluminescence
DMSO: dimethyl sulfoxide
MTT: 3-(4,5-dimethylthiazol-2-yl)-2,5-diphenyltetrazolium bromide
*ΔΨm*: mitochondrial membrane potential
GAPDH: glyceraldehyde-3-phosphate dehydrogenase
SD: standard deviation
IC_50_: 50 % inhibitory concentration
PP2: polyphyllin II
PP6: polyphyllin VI
DCF: dichlorofluorescein
BAD: Bcl2-associated agonist of cell death
STAT3: signal transducer and activator of transcription 3
MAPKs: mitogen-activated protein kinases
JNK: c-JUN N-terminal kinase
ERK: extracellular signal-regulated kinase
p38: p38 MAPK
