# Supplementary figures and images for: Polyphyllin VII induces apoptosis in HepG2 cells through ROS-mediated mitochondrial dysfunction and MAPK pathways
Source: BMC Complement Altern Med. 2016 Feb 9;16:58. doi: 10.1186/s12906-016-1036-x (PMC4746894; doi:10.1186/s12906-016-1036-x)

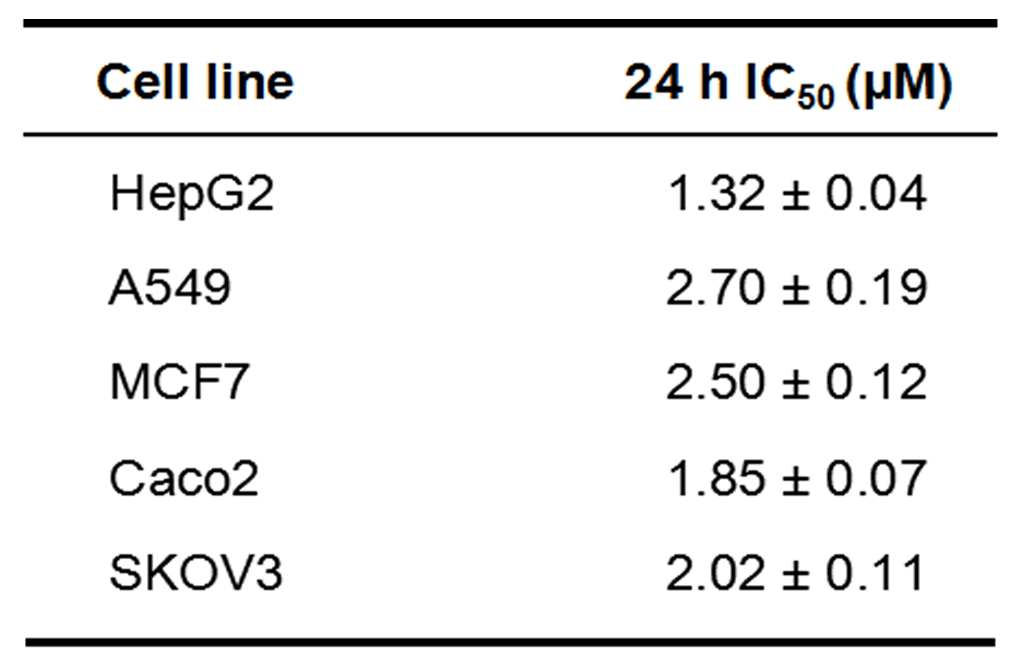

Supplement: Supplementary file 1 — Polyphyllin VII (PP7) inhibited the proliferation of human cancer cells. Cells were treated with increasing concentrations of PP7 for 24 h and cell viability was determined by MTT assay as described in Methods sections. Each IC50 value represents means ± SD of 3 to 5 independent experiments. (TIF 382 kb) [file 12906_2016_1036_MOESM1_ESM.tif]

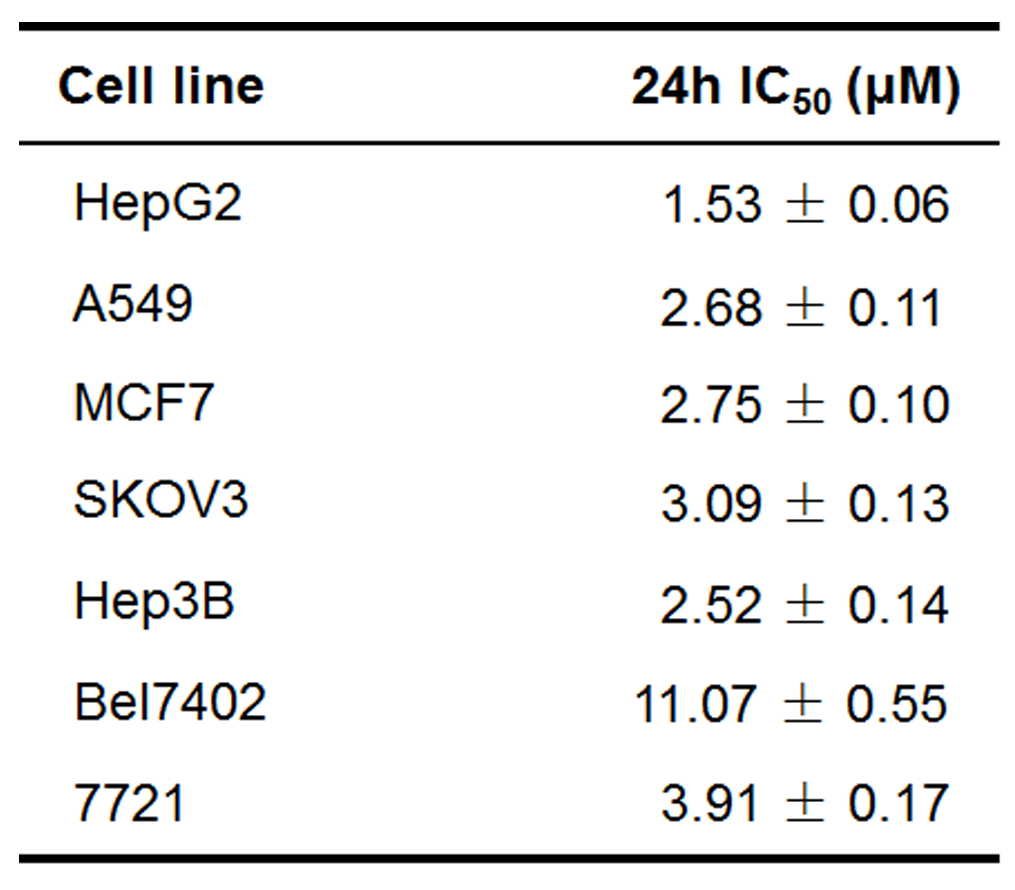

Supplement: Supplementary file 2 — Polyphyllin II (PP2) inhibited the proliferation of human cancer cells (HepG2, A549, MCF7, SKOV3, Hep3B, Bel7402 and 7721). Cells were treated with increasing concentrations of PP2 for 24 h and cell viability was determined by MTT assay as described in Methods sections . Each IC50 value represents means ± SD of 3 to 5 independent experiments. (TIF 593 kb) [file 12906_2016_1036_MOESM2_ESM.tif]

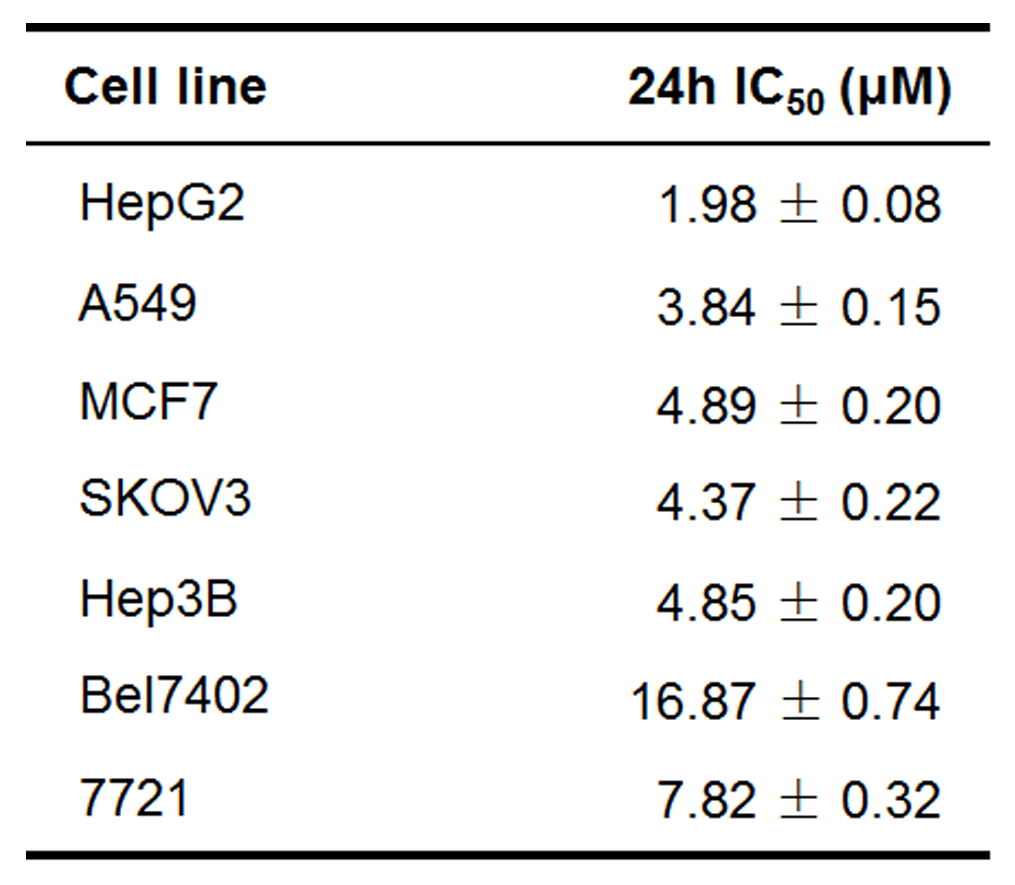

Supplement: Supplementary file 3 — Polyphyllin VI (PP6) inhibited the proliferation of human cancer cells (HepG2, A549, MCF7, SKOV3, Hep3B, Bel7402 and 7721). Cells were treated with increasing concentrations of PP6 for 24 h and cell viability was determined by MTT assay as described in Methods sections. Each IC50 value represents means ± SD of 3 to 5 independent experiments. (TIF 594 kb) [file 12906_2016_1036_MOESM3_ESM.tif]
